# PGF2*α*-FP Receptor Ameliorates Senescence of VSMCs in Vascular Remodeling by Src/PAI-1 Signal Pathway

**DOI:** 10.1155/2022/2908261

**Published:** 2022-01-27

**Authors:** Bo-ang Hu, Wen-wen Sai, Jun Yuan, Hong-tao Lan, Jia Qi, Di Wang, Wei Zhang, Zhi-hao Wang, Ming Zhong, Yuan-yuan Shang

**Affiliations:** ^1^The Key Laboratory of Cardiovascular Remodeling and Function Research, Chinese Ministry of Education, Chinese National Health Commission and Chinese Academy of Medical Sciences, The State and Shandong Province Joint Key Laboratory of Translational Cardiovascular Medicine, Department of Cardiology, Qilu Hospital, Cheeloo College of Medicine, Shandong University, Jinan, Shandong 250012, China; ^2^Department of Dermatology, Shandong Provincial Hospital for Skin Diseases & Shandong Provincial Institute of Dermatology and Venereology, Shandong First Medical University & Shandong Academy of Medical Sciences, Jinan 250022, China; ^3^Department of Emergency, Taian City Central Hospital, Taian 271000, China; ^4^Department of Geriatric Medicine, Qilu Hospital, Cheeloo College of Medicine, Shandong University, Shandong Key Laboratory of Cardiovascular Proteomics, Jinan, Shandong 250012, China; ^5^Department of Cardiology, Zibo Central Hospital, Zibo, Shandong 255000, China

## Abstract

Senescence in vascular smooth muscle cells (VSMCs) is involved in vascular remodeling of aged mice. ProstaglandinF2*α-* (PGF2*α*-) FP receptor plays a critical role in cardiovascular diseases (CVDs), hypertension, and cardiac fibrosis. However, its role in senescence-induced arteriosclerosis is yet to be fully elucidated. In this study, we found that FP receptor expression increased in aged mouse aortas and senescence VSMCs. FP receptor gene silencing can ameliorate vascular aging and inhibit oxidative stress, thereby reducing the expression of PAI-1, inhibiting the activation of MMPs, and ultimately improving the excessive deposition of ECM and delaying the process of vascular fibrosis. FP receptor could promote VSMC senescence by upregulated Src/PAI-1 signal pathway, and inhibited FP receptor/Src/PAI-1 pathway could ameliorate VSMCs aging in vitro, evidenced by the decrease of senescence-related proteins P16, P21, P53, and GLB1 expressions. These results suggested that FP receptor is a promoter of vascular aging, by inducing cellular aging, oxidative stress, and vascular remodeling via Src and PAI-1 upregulation.

## 1. Introduction

With the increase of human life expectancy, deferring or preventing the occurrence of aging and aging-related diseases has become one of the urgent problems to be solved. Aging is the leading risk factor for cardiovascular diseases, diabetes, neurodegenerative diseases, cancer, and metabolic diseases. The crucial point for biological aging is vascular aging. Furthermore, common pathophysiological mechanisms of aging include oxidative stress, mitochondrial dysfunction, chronic low-grade inflammation, genomics homeostasis imbalance, telomere shortening, cell senescence, and epigenetic changes [1]. The main pathological features of vascular aging include changes in lumen dilation, diffuse stiffness, and thickening of the arterial wall [2]. In addition, arteriosclerosis is the most important manifestation of vascular aging. Studies have found that senescence of vascular cells, especially vascular smooth muscle cells (VSMCs), plays an important role in the occurrence and development of arteriosclerosis [3]. By secreting a variety of cytokines and growth factors, aging VSMCs can change the local microenvironment of tissues and promote the occurrence of inflammatory response and vascular sclerosis [4], thereby aggravating the development of atherosclerosis and hypertension. However, the prevention mechanism of VSMC senescence is still lacking. Further study on the relationship between cellular signaling pathways and age-related vascular dysfunction may contribute to a better understanding of vascular aging.

ProstaglandinF2*α* (PGF2*α*) is one of the metabolites of arachidonic acid induced by cyclooxygenase. By binding to the FP receptor, PGF2*α* activates a series of signaling cascades in cells to regulate gene transcription, thus causing corresponding physiological and pathological changes. Recently, more and more attention has been paid to the role of the PGF2*α*-FP receptor in the pathogenesis of cardiovascular diseases. The PGF2*α*-FP receptor is involved in developing atherosclerosis, hypertension, fibrosis, and other age-related diseases. Some studies have shown that PGF2*α* can regulate the expression of renin through FP receptor to regulate blood pressure. Moreover, the PGF2*α*-FP receptor participates in fibrosis by regulating the PKC/Rho kinase pathway in diabetic cardiomyopathy. Notably, the PGF2*α*-FP receptor also plays an active role in the formation of atherosclerosis [5]. Clinical studies have found that the level of PGF2*α* in the elderly is significantly increased as well [6]. Our previous studies have demonstrated that the PGF2*α*-FP receptor is involved in the arteriosclerosis process of diabetic rats. Similarly, the expression of FP receptor in the blood vessels of diabetic rats increased significantly. Silencing the FP receptor gene can attenuate the vascular remodeling of diabetic rats [7]. Therefore, we hypothesized that the FP receptor is involved in the process of senescence-induced vascular sclerosis, but the specific mechanism of aging induced by FP receptor remains unclear.

c-Src, a vital member of the Src tyrosine kinase family, is phosphorylated and activated after stimulation. Activated Src can phosphorylate many cell membrane proteins, cytoplasmic proteins, and nuclear proteins, causing intracellular signal transduction. Studies have revealed that PGF2*α* can activate Src after binding to the FP receptor, which can regulate the activity of Src [8] and cause the proliferation of VSMCs [9]. Indeed, as a senescence marker molecule, PAI-1 can directly act on vascular cells and participate in various biological processes such as cell proliferation, apoptosis, migration, and vascular remodeling. Notably, PAI-1 is a necessary and sufficient role for cell replicative senescence [10], and increased expression of PAI-1 in VSMCs requires Src activation [11]. Therefore, we speculate that FP receptor may promote VSMC senescence through the Src/PAI-1 pathway, leading to vascular senescence.

In this study, we established a natural aging model in mice and observed the effects of FP receptor gene silencing on vascular aging, cellular aging, oxidative stress, and vascular remodeling. We discussed the role of the PGF2*α*-FP receptor/Src/PAI-1 signaling pathway in vascular aging, which provided a theoretical basis for clinical prevention and delay of vascular aging.

## 2. Materials and Methods

### 2.1. Animal Experiment

Twenty-eight six-week-old male C57BL/6J/WT mice were purchased from the Animal Experimental Center of Shandong University. The animals were raised in the animal room of the Cardiovascular Laboratory at Qilu Hospital of Shandong University. Mice were randomly divided into four groups (7 mice in each group): WT young group, WT old group, old+vehicle group, and old+FP receptor shRNA group. After 12 months of age, the lentivirus harboring the FP receptor gene (FP receptor shRNA) and control empty virus (vehicle) was injected via the tail vein in old groups, respectively. Lentiviral transfer was repeated over two weeks. Young and old mice were fed until 3 or 18 months, respectively. At the end of the experiment, the mice were sacrificed. All animal procedures were performed in accordance with the institutional guidelines of Qilu Hospital of Shandong University and approved by the Shandong University Institutional Animal Care and Use Committee.

### 2.2. Cell Culture

Primary rat vascular smooth muscle cells (VSMCs) were extracted from the aortas of SD wild-type rats for 8-10 weeks. In short, the rats were sacrificed, and the aorta was isolated under sterile conditions. After the aorta was chopped, the tissue block was evenly spread at the bottom of the culture bottle, and DMEM with 20% FBS (Gibco, NY, USA) was added. After 2 hours, it was turned over. Cells grew around the tissue block in 3-5 days. The grown cells were subcultured and cultured for the next experiment. Human aortic smooth muscle cells (HASMCs) were obtained from ScienCell Research Laboratories (CA, USA) and cultured in smooth muscle cell medium (SMCM). All cells were cultured in 37°C and 5% CO_2_.

### 2.3. Establishment and Identification of Cell Senescence Model

The primary rat VSMCs were serially passaged for 10 times to establish a replicatively senescence (RS) model. The cells were subcultured 3-5 times and treated with 33.3 mM glucose for 24 hours as the induced senescence (IS) model. HASMCs were also stimulated with 33.3 mM glucose as the IS model. Finally, we passed SA-*β*-Gal staining and Western blot analysis of P16, P21, P53, and GLB1 were used to evaluate the degree of cell senescence.

### 2.4. Ultrasonography

Mice were anesthetized with inhaled anesthetic isoflurane. The chest and abdomen of mice were depilated. Vascular ultrasound (Vevo 770; Visual Sonics) was performed on mice. Intima-media thickness (IMT), peak systolic velocity (PSV), diastolic diameter (Dd), and systolic diameter (Ds) measurements were measured using the effective methods described before. All measures were taken by the same investigator and represented the average of three consecutive cardiac cycles.

### 2.5. Blood Pressure Measurement

The pulse based on tail-cuff method with a photoelectric device was used to measure systolic blood pressure (SBP), diastolic blood pressure (DBP), mean blood pressure (MBP), and heart rate at 3 and 18 months in mice. Blood pressure and heart rate were reported as a mean of 3 consecutive measurements.

### 2.6. Tissue Preparation

After the aorta was detached, it was cleaned in a phosphate buffer solution (PBS). We preserved the upper aorta (about 5 mm) in 4% neutral formaldehyde and then dehydrated in ethanol, embedded in paraffin, and sectioned (5 *μ*m thickness) for histological studies. The remaining aorta was frozen in liquid nitrogen and stored at -80°C for molecular experiments.

### 2.7. Histopathological Analysis

In sections stained with hematoxylin-eosin (Jiancheng Biology Engineering Institute, Nanjing, China), morphological alterations in the aorta walls were found. Sirius red (Abcam ab15081) and Masson (Servicebio GP1032) staining were used to assess the collagen deposition of the aorta walls. Verhoeff (Abcam ab150667) staining was used to determine elastin content. An Olympus DP72 (Olympus, Japan) digital photography device was used to capture the photographs.

### 2.8. Immunohistochemical Analyses

After dewaxing, hydration, endogenous peroxidase activity, and nonspecific binding blocking treatment, the sections were blocked with 5% BSA at 37°C for half an hour and at 4°C overnight incubated with primary antibody: Anti-FP receptor (Cayman 101802), Anti-CollagenI (Abcam ab34710), Anti-CollagenIII (Abcam ab7778), Anti-MMP2 (Abcam ab97779), and Anti-MMP9 (Abcam ab76003). The slices were washed three times with PBS and incubated with secondary antibodies at 37°C for 50 minutes. The bound secondary antibodies was detected using DAB solution (Zhongshan Jinqiao Biotechnology, Beijing, China). The reactions were observed under a microscope and stopped in distilled water when target area turned yellow. Lastly, the nuclei were stained with hematoxylin, and the slides were dehydrated, cleared, and mounted. The images were acquired using an Olympus DP72 digital imaging system and analyzed using Image Pro Plus (IPP) 6.0 software.

### 2.9. Western Blot Analysis

The cells or the artery tissues were added into the 150 *μ*L RIPA containing PMSF (RIPA: PMSF = 100 : 1), placed on ice to split 20-30 min, and centrifuged at 4°C (12000 rpm 10 min), and the supernatant was collected. The protein concentration was detected by the BCA (Solarbio PC0020, China) method, and the samples were detected by SDS-polyacrylamide gel electrophoresis. The sample concentration per hole was 30 *μ*g, and then, the gel was transferred to the PVDF membrane. The membranes were blocked in 5% nonfat dried milk/Tween 20-tris buffered saline (TBST) for 1 h and incubated overnight at 4°C with a primary antibody, including Anti-FP receptor (Cayman 101802), Anti-Src (CST 36D10), Anti-p-Src (CST D49G4), Anti-PAI-1 (NOVUS 19773), Anti-P16 (Proteintech 10883-1-AP), Anti-P21 (Abcam ab109199), Anti-P53 (Abcam ab26), Anti-GLB1 (Abcam ab128993), Anti-eNOS (Abcam ab76198), and Anti-p-eNOS (CST C9C3). After three cycles of cleaning with TBST, membranes were incubated in appropriate horseradish peroxidase-conjugated secondary antibodies and observed by enhanced chemiluminescence (ECL) reagent (Millipore WBKLS0500). Image J software was used for analysis. All experiments were performed at least three times.

### 2.10. Detection of Nitric Oxide and Reactive Oxygen Species

Nitric oxide (NO) was measured by NO-sensitive fluorescent dye (DAF-FM) (Bestbio BB-46083, Shanghai, China). Reactive oxygen species (ROS) was measured using DHE (Bestbio BB-470516, Shanghai, China). For both fluorescent dyes, recordings were made using the upright fluorescent microscope (Nikon Eclipse 80i) in the en face endothelial side up, probe labeled aortic tissue. Fluorescent data were analyzed using NIS-ELEMENTS Basic Research.

### 2.11. MDA and SOD Level Measurement

MDA and SOD levels in serum were analyzed by using commercially available kits (Jiancheng Biology Engineering Institute, Nanjing, China).

### 2.12. Determination of Telomere Length

Genomic DNA of VSMCs was extracted by DNA extraction kit (Sparkjade AA1004, Jinan, China). After stimulating, cells were treated into single-cell suspension, and supernatant was discarded after centrifugation. 180 *μ*L PBS+20 *μ*L protease K (20 mg/mL) were added and mixed evenly. 200 *μ*L CB has added again and placed at 70°C for 10 min. 100 *μ*L isopropyl alcohol was added after cooling. The mixture was added to AC adsorption column for centrifugation, and the waste liquid was discarded. 500 *μ*L inhibitor removal solution IR was added, and bleaching solution WB was added for cleaning twice after centrifugation. Finally, 50 *μ*L eluting buffer EB was added to collect the solution into EP tube. The concentration of DNA in each sample was detected by Thermo NANO DROP ONE instrument and diluted into 50 ng/*μ*L. The CT values of telomere and single copy gene 36B4 were measured by RT-PCR. The data was collected by the Bio-Rad iQ5 system. The relative ratio of telomere/single copy gene 36B4 = 2^−ΔΔCT^. The primer sequences were as follows:

Tel: forward primer 5′-CGGTTTGTTTGGGTTTGGGTTTGGGTTTGGGTTTGGGTT-3′; reverse primer 5′-GGCTTGCCTTACCCTTACCCTTACCCTTACCCTTACCCT-3′; 36B4: forward primer 5′-ACTGGTCTAGGACCCGAGAAG-3′; reverse primer 5′-TCAATGGTGCCTCTGGAGATT-3′.

### 2.13. Determination of Telomerase Activity

Total RNA was extracted with Trizol reagent (Sparkjade AC010, Jinan, China) and concentration of sample RNA was detected by a spectrophotometer. The reverse transcription kit (Sparkjade AG0304, Jinan, China) was used to reverse 1 *μ*g RNA to cDNA. The real-time fluorescence quantitative PCR telomerase detection kit (KeyGen BioTech KGA1028R) was used to configure the qPCR mix 25 *μ*L system according to the instructions. TRET and GAPDH genes were amplified by two steps: 95°C for 10 min, predenatured, 95°C for 15 s, annealed at 60°C and extended for 1 min, and amplified for 40 cycles. The relative expression of telomerase activity mRNA in each group was calculated according to the CT value. The primer sequences were as follows:

TRET: forward primer 5′-GACATGGAGAACAAGCTGTTTGC-3′; reverse primer 5′-ACAGGGAAGTTCACCACTGTC-3′; GAPDH: forward primer 5′-AGGTTGTCTCCTGTGACTTCAA-3′; reverse primer 5′-CTGTTGCTGTAGCCATATTCATTG-3′.

### 2.14. Senescence-Associated *β*-Galactosidase Staining

The cells and tissues were washed with PBS solution 3 times, 5 min each time, and then treated with stationary solution at room temperature for 15 min. All samples were added with the staining solution according to the instructions of the SA-*β*-Gal staining kit (CST 9860). The samples were incubated at 37°C without CO_2_ to maintain a pH of 6.0. In positive cells, blue-green granules were present in their cytoplasm. For each group, six slice areas were randomly selected, and the positive staining rate was calculated using IPP.

### 2.15. Statistical Analyses

Data are represented as mean ± SD. SPSS 20.0 and GraphPad Prism 6.0 were used for statistical analysis and image presentation. Comparisons among four groups were performed using one-way ANOVA and Tukey test and comparisons between two groups were performed using *t*-tests. *P* < 0.05 was considered statistically significant.

## 3. Results

### 3.1. Establishment of the Natural Aging Mouse Model

A natural aging animal model was established by raising C57 WT mice until they were 18 months old. Western blot showed that the relative contents of the age-related markers P16, P21 and P53 in aortic vessels were significantly higher in old mice than in young mice (Supplementary Figure 1a). The SA-*β*-Gal staining rate was higher in the old group compared with the young group (Supplementary Figure 1c). These results suggested that we had successfully established a natural aging mouse model.

### 3.2. FP Receptor Was Increased in the Aorta of Aging Mice, and FP Receptor Gene Silencing Can Delay Cell Senescence Process in the Aorta of Aging Mice

**Figure 1:**
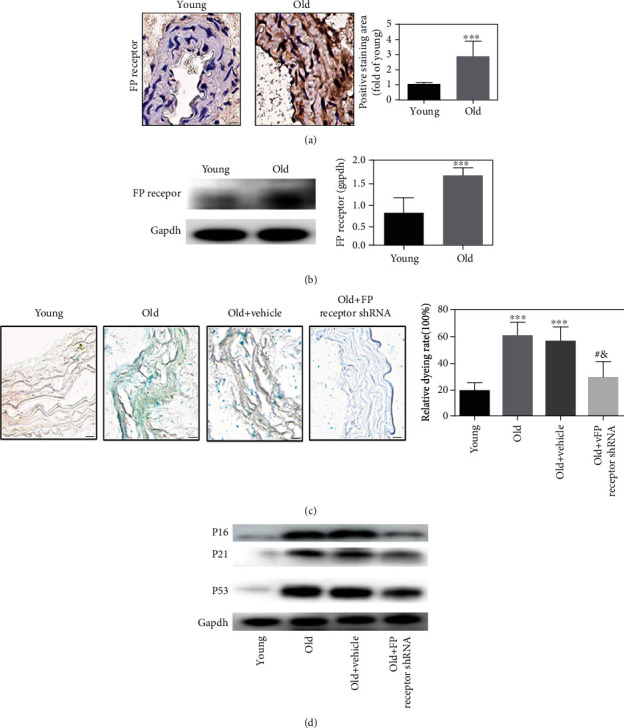
The expression of FP receptor was increased in the aorta of aging mice, and FP receptor gene silencing could delay cell senescence process in the aorta of aging mice. (a) Immunohistochemical staining and analysis of FP receptor. (b) Representative Western blot and analysis of FP receptor. (c) Representative SA-*β*-Gal staining with the aorta from the mice (scale bar: 20 *μ*m). (d) Representative Western blot and analysis of P16, P21, and P53.

Immunohistochemical staining and Western blot results showed that FP receptor expression was significantly higher in old mice than in young mice (Figures 1(a) and 1(b)). These results suggested that the expression of FP receptor in the aortas of aging mice is significantly increased. FP receptor may be involved in the process of vascular aging. To evaluate whether FP receptor could play a role in vascular aging, we detected the relevant indicators of senescence. As excepted, the SA-*β*-Gal staining rate was higher in the old group compared with the young group (Figure 1(c)). With FP receptor-shRNA treatment, the SA-*β*-Gal staining rate was lower in the old+FP receptor-shRNA group compared with the old+vehicle group (*P* < 0.05). Indeed, the results of Western blot showed that the expressions of senescence-associated proteins P16, P21, and P53 were markedly upregulated in the old group compared with the young group (Figure 1(d)). With FP receptor silencing, the expressions of P16, P21, and P53 in the old+FP receptor-shRNA group were significantly decreased compared with the old+vehicle group (Figure 1(d)). These results indicated that FP receptor is involved in senescence process, and FP receptor gene silencing can delay the aging of mouse aortic.

### 3.3. The Role of FP Receptor in Structural Changes of Aging Vessel

**Figure 2:**
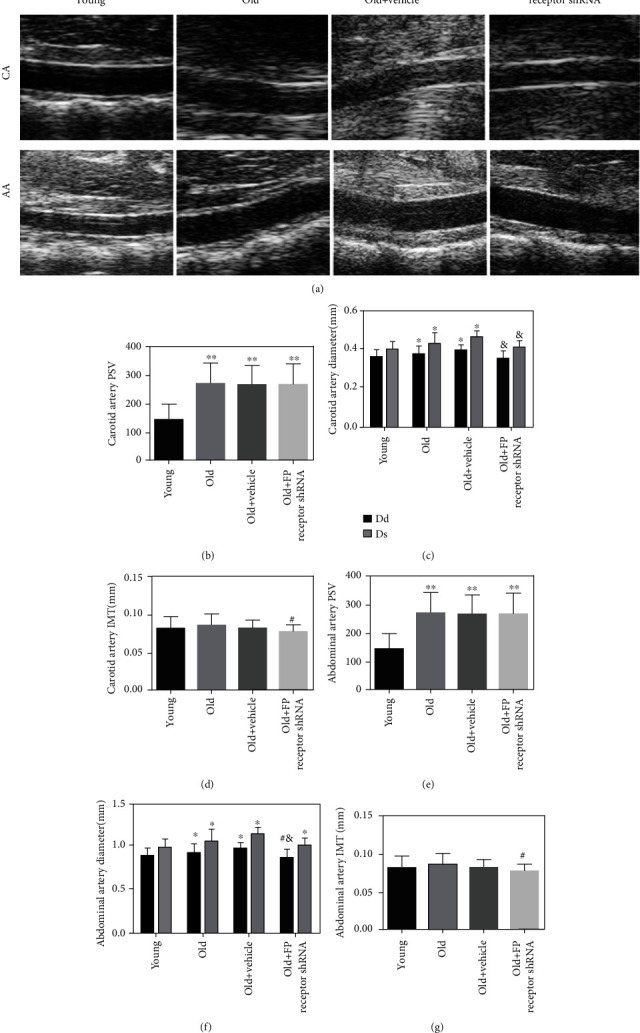
The role of FP receptor in structural changes of aging vessel. (a) CA and AA of ultrasonography. (b) PSV of the carotid artery; (c) Dd and Ds of the carotid artery. (d) IMT of the carotid artery. (e) PSV of the abdominal aorta. (f) Dd and Ds of the abdominal aorta. (g) IMT of the abdominal aorta. ^∗^*P* < 0.05 and ^∗∗^*P* < 0.01 vs. young group; ^#^*P* < 0.05 vs. old group; ^&^*P* < 0.05 vs. old+vehicle group; *n* = 7.

Both diastolic diameter (Dd) and systolic diameter (Ds) of the carotid artery and abdominal aorta in the old group were significantly increased compared with the young group (Figures 2(c) and 2(f)). The peak systolic velocity (PSV) of the carotid artery and abdominal aorta in the old group was higher than in the young group (Figures 2(b) and 2(e)). With FP receptor-shRNA treatment, the intima-media thickness (IMT), Ds, and Dd were decreased in the old+FP receptor-shRNA group compared with the old+vehicle group (Figures 2(c), 2(d), 2(f), and 2(g)). The IMT was significantly increased in the carotid artery and abdominal artery in the old+vehicle group compared with the old+FP receptor shRNA group (Figures 2(d) and 2(g)). However, the IMT of the carotid artery and abdominal aorta in the young group was not significantly thicker than that in the old group (*P* > 0.05). The above results indicated that aging mice have an obvious vascular remodeling. FP receptor gene silencing could delay vascular remodeling in aging mice.

### 3.4. The Role of FP Receptor in Blood Pressure

**Figure 3:**
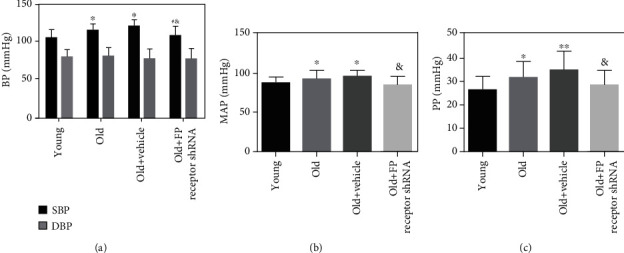
The role of FP receptor in blood pressure of aging vessels: (a) systolic pressure (SBP) and diastolic blood pressure (DSP); (b) mean arterial pressure (MAP); (c) pulse pressure (PP).

Systolic pressure (SBP), mean arterial pressure (MAP), and pulse pressure (PP) were increased in the old group compared with the young group (Figures 3(a)–3(c)). There was no significant difference in diastolic blood pressure (DBP) among all groups (*P* > 0.05). With FP receptor silencing, the SBP, MAP, and PP of the old+FP receptor shRNA group were significantly decreased compared with the old+vehicle group (Figures 3(a)–3(c)).

### 3.5. FP Receptor Gene Silencing Ameliorated Vascular Fibrosis in Aging-Related Arterial Stiffness

**Figure 4:**
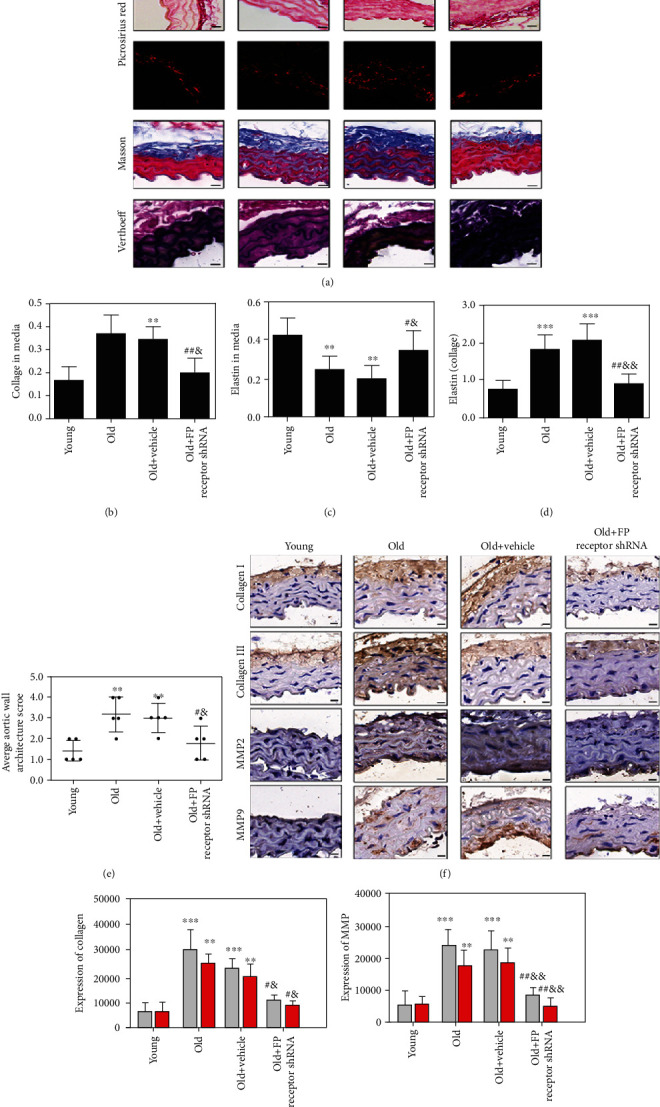
FP receptor gene silencing ameliorated vascular fibrosis in aging-related arterial stiffness. (a) Representative H&E staining, Picrosirius red staining, Masson trichrome staining, and Verhoeff Van Gieson (scale bar: 20 *μ*m). (b–e) Analysis of collagen content, elastin content, collagen content/elastin content, and average aortic wall architecture score. (f) Representative immunohistochemical staining for Collagen I, Collagen III, MMP2, and MMP9. (g, h) Immunohistochemical staining analyses of Collagen I, Collagen III, MMP2, and MMP9. ^∗^*P* < 0.05, ^∗∗^*P* < 0.01, and ^∗∗∗^*P* < 0.001 vs. young group; ^#^*P* < 0.05 and ^##^*P* < 0.01 vs. old group; ^&^*P* < 0.05 and ^&&^*P* < 0.01 vs. old+vehicle group; *n* = 5.

The Masson trichrome staining, Picrosirius red staining, and Verhoeff's Van Gieson staining results showed vascular fibrosis in the media. The collagen contents, collagen-to-elastin ratio, and average aortic wall architecture score were significantly increased, and elastin content was decreased in the old group compared with that in the young group (Figures 4(a)–4(e)). With FP receptor-shRNA treatment, the collagen content, collagen-to-elastin ratio, and average aortic wall architecture score were markedly decreased, and elastin content was increased in the old+FP receptor shRNA group compared with that in the old+vehicle group (Figures 4(a)–4(e)). The above results suggested that there are obvious structural disorders and fibrosis of aortic vessels in aging mice. FP receptor gene silencing could reverse vascular structural disorders and fibrosis in aging mice. Indeed, FP receptor gene silencing ameliorated vascular fibrosis in aging-related arterial stiffness. Immunohistochemical staining results showed that Collagen I, Collagen III, MMP2, and MMP9 expressions were significantly increased in the old group compared with that in the young group (Figures 4(f)–4(h)). The Collagen I, Collagen III, MMP2, and MMP9 expressions in the old+FP receptor shRNA group were lower than those in the old+vehicle group (Figures 4(f)–4(h)).

### 3.6. Effects of FP Receptor on NO Level and Oxidative Stress in the Aorta of Aging Mice

**Figure 5:**
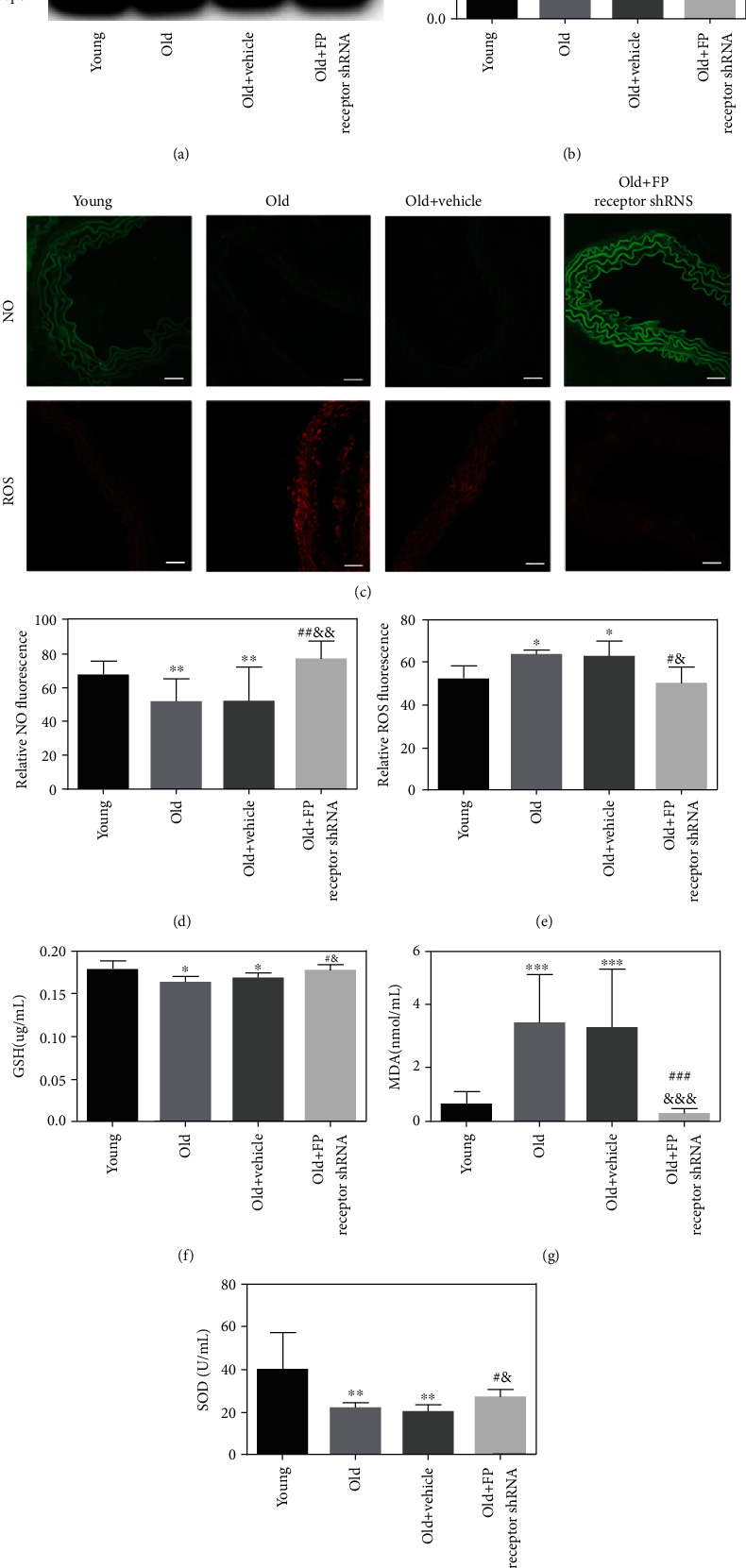
Effect of FP receptor on vascular oxidative stress in aging mice. (a) Representative Western blot of p-eNOS and t-eNOS; (b) Western blot analysis of figure (a). (c) Representative DAF-FM and DHE staining with the aorta from the mice (scale bar: 20 *μ*m). DAF-FM and DHE to detect the expressions of NO and ROS in four groups of mice (scale bar: 20 *μ*m). (d, e) Analysis of the relative dyeing rate. (f–h) The levels of MDA, GSH, and SOD in the serum of four groups. ^∗^*P* < 0.05, ^∗∗^*P* < 0.01, and ^∗∗∗^*P* < 0.001 vs. young group; ^#^*P* < 0.05, ^##^*P* < 0.01, and ^###^*P* < 0.001 vs. old group; ^&^*P* < 0.05, ^&&^*P* < 0.01, and ^&&&^*P* < 0.001 vs. old+vehicle group; *n* = 5.

Western blot showed that the relative protein expression of p-eNOS/t-eNOS in the old group was significantly lower than that in the young group (Figures 5(a) and 5(b)). Treatment with FP receptor-shRNA increased the p-eNOS/t-eNOS expression of aging mice (Figures 5(a) and 5(b)). The levels of GSH, MDA, and SOD in the serum of mice were detected by assay kits. The GSH and SOD levels were decreased, and the MDA level was increased in the old group compared with that in the young group (Figures 5(f)–5(h)). In addition, FP receptor gene silence also decreased the MDA level and increased the SOD and GSH levels in the serum of aging mice (Figures 5(f)–5(h)). DAF-FM and DHE staining showed that ROS expression was upregulated and NO expression was downregulated in the old group compared with that in the young group (Figures 5(c)–5(e)). However, FP receptor gene silencing downregulated ROS expression and upregulated NO expression in the thoracic aorta of aging mice. There was a statistically significant difference in ROS and NO expression between the FP receptor shRNA and the old group (Figures 5(c)–5(e)). The above results suggested that there is obvious oxidative stress in the aorta of aging mice. FP receptor gene silencing can downregulate the level of oxidative stress in aging mice.

### 3.7. The Decreased Expressions of p-Src and PAI-1 Were Related to the Protective Effect of FP Receptor Gene Silencing on Vascular Aging

**Figure 6:**
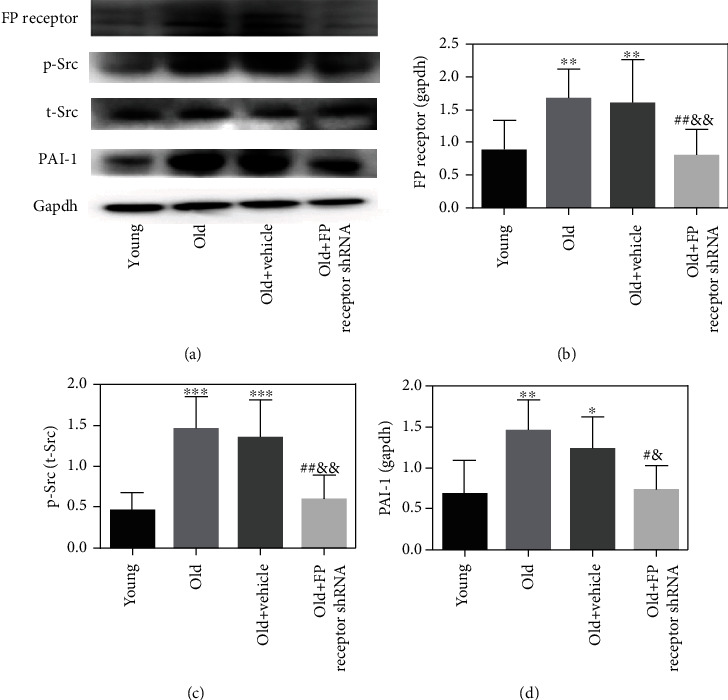
Effect of FP receptor gene silencing on the expressions of p-Src and PAI-1 in the aorta of aging mice. (a) Representative Western blot of FP receptor, p-Src, t-Src, and PAI-1 expressions. (b–d) Western blot analysis of FP receptor, p-Src/t-Src, and PAI-1. ^∗^*P* < 0.05, ^∗∗^*P* < 0.01, and ^∗∗∗^*P* < 0.001 vs. young group; ^#^*P* < 0.05 and ^##^*P* < 0.01 vs. old group; ^&^*P* < 0.05 and ^&&^*P* < 0.01 vs. old+vehicle Group; *n* = 5.

Western blot results showed the protein of FP receptor, p-Src, and PAI-1 expressions were significantly higher in the old group compared with that in the young group (Figures 6(a)–6(d)). After FP receptor gene silencing, the expressions of FP receptor, p-Src, and PAI-1 were markedly decreased in the old+FP receptor-shRNA group compared to those in the old+vehicle group (Figures 6(a)–6(d)).

### 3.8. PGF2*α*-FP Receptor Was Involved in the VSMC Senescence Process

**Figure 7:**
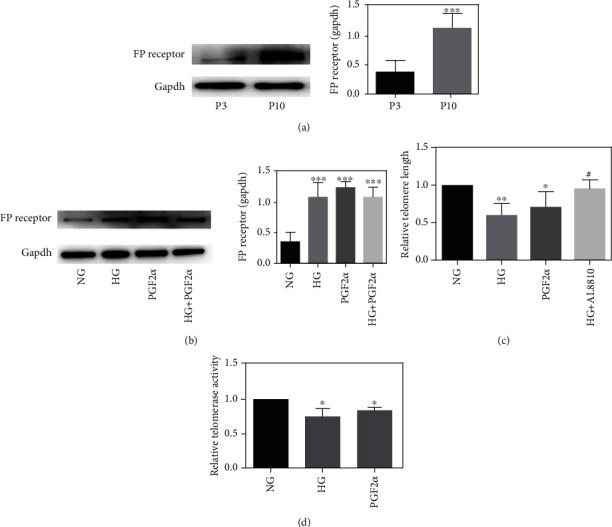
(a) The expression of FP receptor protein in naturally aging VSMCs. (b) The effect of HG and PGF2*α* on the expression of FP receptor protein. (c) Telomere length was detected by RT-PCR. (d) Telomerase activity was detected by RT-PCR.

In order to further verify that the PGF2*α*-FP receptor promotes the senescence of VSMCs, we cultured primary rat VSMCs and established a replicating senescence model through the natural continuous passage method (Supplementary Figure 2a-b). Western bolt results showed that compared with the young group (P3), the protein expression level of FP receptor in the natural aging group (P10) was significantly increased (*P* < 0.05) (Figure 7(a)). After 24 h stimulation with PGF2*α*, compared with the control group, the protein expression of the FP receptor in the PGF2*α* group was significantly increased, while the telomere length and telomerase activity were decreased markedly (Figures 7(b)–7(d)). The results indicated that the PGF2*α*-FP receptor was involved in the aging process of VSMCs. With FP receptor inhibitor AL8810 treatment, the telomere length was significantly extended (Figure 7(c)). Meanwhile, high glucose (HG) and PGF2*α* can decrease telomerase activity in VSMCs (Figure 7(d)). The same results were verified in HASMCs as well. Both HG and PGF2*α* stimulation could increase the expression of FP receptor in HASMCs and elevate the aging-related protein expressions of P16, P21, and GLB1 (Supplementary Figure 4). The above results indicated that HG and PGF2*α* can promote the senescence of VSMCs. PGF2*α*-FP receptor is involved in the senescence process of VSMCs induced by HG.

### 3.9. The Role of PGF2*α*-FP Receptor/Src/PAI-1 Signaling Pathway in VSMC Senescence

**Figure 8:**
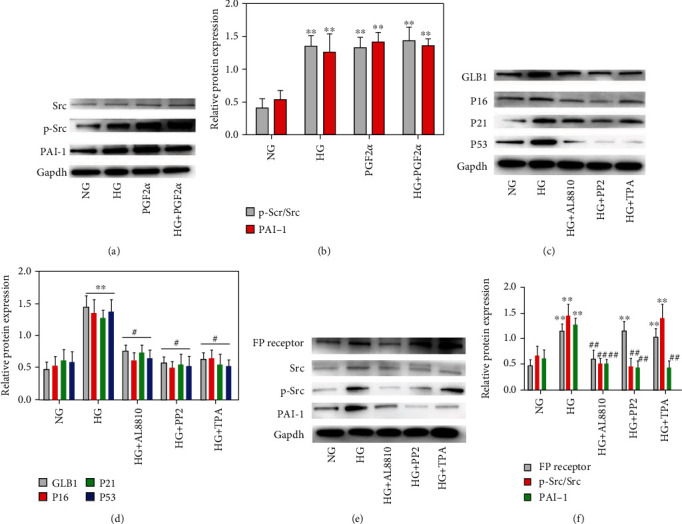
(a) Western blot of Src, p-Src, and PAI-1 protein expression levels under different conditions. (b) Quantitative analysis of figure (a). (c) Western blot of GLB1, P16, P21, and P53 expression levels under different conditions. (d) Quantitative analysis of figure (c). (e) Western blot of FP receptor, Src, p-Src, and PAI-1 protein expression levels under different conditions. (f) Quantitative analysis of figure (e).

VSMCs were stimulated with PGF2*α* and HG, respectively. Western blot showed that compared with the control group, the p-Src and PAI-1 protein expression levels of VSMCs in the HG group were significantly increased (Figure 8(a)) (both *P* < 0.05), while the total Src level was not significantly changed (Figure 8(a)) (*P* > 0.05). The results were the same in the PGF2*α* group. When HASMCs were stimulated by HG and PGF2*α*, the expressions of p-Src and PAI-1 increased compared with the NG group (Supplementary Figure 5). These results suggested that HG may induce senescence of primary rat VSMCs and HASMCs through the PGF2*α*-FP receptor/Src/PAI-1 signaling pathway. In order to further clarify the role of the PGF2*α*-FP receptor/Src/PAI-1 signaling pathway in the IS model of VSMCs, VSMCs were divided into five groups: NG group, HG group, HG+FP receptor inhibitor AL8810 group, HG+Src inhibitor PP2 group, and HG+PAI-1 inhibitor TPA group. The results showed that compared with the HG group, the expression levels of cell senescence-related indicator proteins GLB1, P16, P21, and P53 in the HG+AL8810 group, the HG+PP2 group, and the HG+TPA group were significantly decreased (Figure 8(c)). Moreover, in the IS model of HASMCs, the senescence-related protein expressions of GLB1, P16, and P21 lower markedly and the positive rate of *β*-galactosidase staining decreased significantly after AL8810 stimulation (Supplementary Figure 6). In conclusion, HG can promote the senescence of VSMCs through the FP receptor/Src/PAI-1 signaling pathway, and inhibition of this pathway can delay the senescence of VSMCs.

Next, Western blot showed that in the IS model, after AL8810 intervention, the expressions of FP receptor, p-Src, and PAI-1 decreased significantly in the HG group (Figure 8(e)) (*P* < 0.05), but the total Src level did not change significantly (*P* > 0.05). Under the condition of HG in HASMCs, the intervention of AL8810 can also lead to the decrease of p-Src and PAI-1 (Supplementary Figure 6). After intervention with PP2, a specific inhibitor of Src, the expressions of p-Src and PAI-1 in the HG+PP2 group were significantly reduced compared with the HG group (Figures 8(e) and 8(f)), but the FP receptor and total Src levels did not change significantly (Figures 8(e) and 8(f)). After the intervention of TPA, the expression of PAI-1 in the HG+TPA group was significantly reduced compared with that in the HG group (Figures 8(e) and 8(f)), but the levels of FP, p-Src, and total Src did not change significantly (Figures 8(e) and 8(f)). Considering the above results, we conclude that the FP receptor/Src/PAI-1 signaling pathway is involved in the senescence of VSMCs, and the PGF2*α*-FP receptor is the critical factor in this pathway.

## 4. Discussion

In this study, we revealed that aortas of aged mice show signs of vascular remodeling, changes in vasoconstriction, and diastolic function changes along with increased matrix metalloproteinases, collagen fibers, and other extracellular matrix and accompanied by the high expression of FP receptor. Furthermore, FP receptor promotes the senescence of VSMCs by activating the Src/PAI-1 signaling pathway, which leads to vascular senescence. On the other hand, FP receptor gene silencing can slow down the aging process of VSMCs and ameliorate vascular remodeling. Therefore, the FP receptor could be an effective therapeutic candidate against vascular aging and is expected to delay aging.

Vascular senescence refers to the structural and functional changes of blood vessels due to aging. It can be divided into pathological vascular senescence caused by various diseases and physiological vascular senescence caused by aging, characterized by increased vascular stiffness, decreased compliance, and arterial wall thickening. Vascular aging is accompanied by increased collagen synthesis and altered elastic fiber properties. In the process of aging, the area of collagen bundle increased significantly, while the amount and circumference of collagen decreased. The abnormal distribution of fibers, elastase activity, and calcium-binding elastin were raised with the change of elastin quality and quantity. In this study, we also observed that the positive staining rates of aging-related proteins P16, P21, P53, and *β*-galactosidase in the aorta of elderly mice were significantly increased, and SBP, MAP, PP, PSV, DS, DD, IMT, collagen-elastin ratio, and mean aortic wall structure score were all increased, as well as the expressions of Collagen I, Collagen III, and MMPs. In addition, the aged mice had elevated blood pressure, dilated lumens, thickened walls, and increased in dimensionality. These results further confirm the existence of significant vascular aging in the aorta of aging mice.

PGF2*α*, an inflammatory mediator, plays a role through the FP receptor (one of the G protein-coupled receptors). FP receptor is expressed in vascular endothelial cells and vascular smooth muscle cells [12]. Activation of FP receptor triggers signal transduction by increasing intracellular calcium and inositol phosphate accumulation [8]. Studies have shown that PGF2*α*-FP receptor can modulate extracellular matrix and vascular function [13]. Meanwhile, PGF2*α*-FP receptor also has a meaningful impact in the development and progression of cardiovascular events. For instance, PGF2*α* could increase blood pressure in rodents, and FP receptor antagonists could treat hypertension and associated vascular diseases [14]. Knockout of FP receptor delayed atherosclerosis in hyperlipidemic mice [5]. Currently, there is little available information about the relationship between aging and PGF2*α*-FP receptor. Moreover, it was found that the level of PGF2*α* mRNA in the aorta of aged rats was two times higher than that of young rats [15]. Therefore, we speculate that PGF2*α*-FP receptor may be involved in the process of vascular aging. We manifest that the expression of FP receptor in the aorta of aging mice increased significantly. After FP receptor gene silencing, the vascular hardness, collagen content, collagen/elastin ratio, average aortic wall structure score, and MMP content decreased meaningfully. These results suggest that FP receptor gene silencing delays aging-related vascular remodeling.

Aging is a major risk factor for hypertension, and the prevalence rate of hypertension increases with age. Previous studies have confirmed that aging can affect blood pressure by regulating endothelin synthesis, NO activity, baroreflex, and inflammatory reaction. It is manifested as increased systolic blood pressure, unchanged or slightly decreased diastolic blood pressure, increased pulse pressure difference, and large blood pressure variability. Our study found that SBP, PP, and MAP of aging mice increased significantly, but the change of DBP was not obvious, which was consistent with the characteristics of hypertension in the elderly in clinical studies. After FP receptor gene silencing, the SBP, PP, and MAP were significantly lower. These results suggest that FP receptor gene silencing may ameliorate age-induced hypertension. The PGF2*α*-FP receptor has been reported to be positively associated with increased blood pressure [5]. FP receptors have been shown to affect the renin-angiotensin-aldosterone system (RAAS). In addition, there is an interaction between PGF2*α*-FP receptor and AT1R that enhances AngII-induced vasoconstriction [16]. In rats, PGF2*α*-FP receptor can increase Ca^2+^ concentration in vascular smooth muscle cells and increase vascular pressure [17]. However, in JG cells, the stimulation of PGF2*α* did not cause a significant increase in renin [5]. PGF2*α* also regulates blood pressure through TP receptor. Therefore, the relationship between PGF2*α*-FP receptor and blood pressure needs to be further explored.

Mitochondrial dysfunction in aging will increase the production of ROS, and the decreased aortic compliance caused by increased oxidative stress is mainly caused by VSMC-mediated vascular wall remodeling [18]. The increase of c-Src activity can mediate the increase of ROS production, which can impair vascular function. Activated c-Src can upregulate NOX1-mediated ROS production by activating and membrane-localizing small GTPase Rac-1 [19]. Another study indicated that high levels of prostaglandins synthesized by COX-2 increased ROS production through the c-Src/NADPH oxidase pathway. In addition, selective c-Src inhibitors could inhibit ROS production, NADPH oxidase activity, and p47phox translocation [20]. Our results showed that the expression of p-eNOS and NO in the aorta of aging mice was significantly reduced, the levels of ROS were markedly increased, the activity of GSH and SOD was decreased, and the oxidation index MDA was increased. FP receptor gene silencing restored the increased of p-Src, MDA, and ROS, and decreased of p-eNOS, GSH, SOD, and NO in aging aorta. The above results suggest that FP receptor gene silencing can improve the oxidative stress response caused by aging. As one of the direct effect proteins of membrane receptors, c-Src can receive and transmit signals from membrane receptors. Therefore, c-Src may act as a direct effector protein of FP receptor to mediate the generation of oxidative stress response, thus causing arteriosclerosis.

Aging can lead to excessive arterial fibrosis and extracellular matrix deposition and aggravate aging-related vascular damage and stiffness [21]. At the molecular and cellular levels, one of the hallmarks of arterial aging is reduced NO production, increased ROS production (oxidative stress), activating of transcription factors, and inducing of expression of aging-related genes. This further stimulates the activation of proinflammatory and fibrosis signaling pathways, leading to deterioration of vascular function. The biomechanical properties of blood vessels are determined by the content of collagen and elastin, so excessive collagen deposition and loss of elastin can lead to vascular stiffness and fibrosis [22]. Proinflammatory factors, growth factors, vasoactive substances, and ROS can trigger the activation of ECM proteins (including collagen and elastin), and ECM is also regulated by the endopeptidase family MMPs.

Activated MMPs induce phenotypic transformation of smooth muscle cells and endothelial cells, which contributes to fibrosis and further aggravates vascular remodeling and arterial stiffness. Plasminogen activator inhibitor 1 (PAI-1), a member of the serine protease inhibitor (SERPIN) gene family, can inhibit fibrinolysis. Indeed, PAI-1 inhibits the degradation of ECM by reducing the production of plasmin and maintains tissue homeostasis by regulating the activity of MMPs. Under pathological conditions, PAI-1 reduces collagen degradation and aggravates ECM protein accumulation and tissue fibrosis. The activity and expression of PAI-1 were also significantly increased in aging experimental models and elderly individuals [23]. Our research found that the production of ROS, the secretion of collagen, and the expression of MMPs in the aorta of aged mice increased, while the expression of NO and the content of elastin decreased. FP receptor gene silencing may improve vascular aging and inhibit oxidative stress, thereby reducing the expression of PAI-1, inhibiting the activation of MMPs, and ultimately improving the excessive deposition of ECM and delaying the process of vascular fibrosis.

Cellular senescence is the basis of tissue senescence and the fundamental cause of vascular senescence, which plays an important role in the formation of arteriosclerosis [4]. Senescent cells are highly expressed with negative regulatory factors of cell cycle, such as P53 and P16 [24]. As a key mediator in cell senescence, the expression of PAI-1 increased in a time-dependent manner in natural senescence cell models and in induced senescence models [25]. Another study found that PAI-1 may induce senescence by preventing t-PA-mediated proteolysis of IGFBP3 [26]. Generally speaking, PAI-1 modulates cell senescence by regulating the extracellular proteolysis of SASP, which acts as the direct and decisive factor of cell senescence in different cell types. In order to explore the role of the FP receptor/Src/PAI-1 pathway in cell senescence, we detected the related indexes of cell senescence. We found that the expressions of FP receptor, p-Src, PAI-1, *β*-galactosidase staining, and senescence-related proteins P16, P21, P53, and GLB1 were significantly increased in aging aortic vessels. The above results indicate that cellular senescence is involved in vascular senescence. The expressions of p-Src and PAI-1, the expressions of P16, P21, and P53, and the positive rate of *β*-galactosidase staining were decreased after FP receptor gene silencing, which indicated FP receptor gene silencing improved cell senescence.

In order to further verify the effect of FP receptor on senescence of VSMCs, we established the model of natural aging and induced aging of VSMCs and found that the expressions of FP receptor, p-Src, and PAI-1 in aging VSMCs all increased significantly. Both in primary rat VSMCs and HASMCs, we used PGF2*α* to stimulate, aging-related proteins were significantly increased, and telomerase activity was significantly reduced. To further prove the role of PGF2a-FP receptor/Src/PAI-1 in VSMCs senescence, we first used PGF2*α* to stimulate VSMCs and found that the expressions of p-Src and PAI-1 in VSMCs were significantly increased, considering that Src/PAI-1 is the downstream signal molecule of PGF2*α*-FP receptor. In the IS model of VSMCs, we found that AL8810, an inhibitor of FP receptor, significantly inhibited the expressions of p-Src and PAI-1. Then, we applied the Src inhibitor PP2 and found that the expression of PAI-1 was significantly reduced, while the expression of FP receptor was not significantly affected. After administration of PAI-1 inhibitor tPA, the expression levels of FP receptor and p-Src were not affected. When in the IS model, FP receptor inhibitor AL8810, Src inhibitor PP2, and PAI-1 inhibitor TPA were given, respectively, we found that the expressions of senescence-related proteins decreased. Therefore, it is speculated that Src/PAI-1 is the downstream effector of FP receptor. FP receptor can promote primary rat VSMCs and HASMC senescence by upregulating the Src/PAI-1 signal pathway, and the FP receptor/Src/PAI-1 pathway inhibitor can ameliorate rat and human VSMCs aging.

## 5. Conclusions

The PGF2*α*-FP receptor is a promoter of vascular aging, by inducing cellular aging, oxidative stress, and vascular remodeling via direct Src and PAI-1 upregulation, respectively. Our results indicate that the PGF2*α*-FP receptor may be an attractive target for age-related cardiovascular diseases.

## Data Availability

The data that support the findings of this study are available from the corresponding author upon reasonable request.

## Supplementary Materials

Supplementary Materials Supplementary Figure 1: establishment of natural aging mouse model. WT C57 mice were divided into young and old groups. (a) Representative Western blot of P16, P21, and P53; (b) Western blot analysis of P16, P21, and P53; (c) Representative *β*-galactosidase staining with the aorta from the mice (scale bar: 20 *μ*m); (d) Analysis of the relative dyeing rate. ^∗^*P* < 0.05, ^∗∗^*P* < 0.01, and ^∗∗∗^*P* < 0.001 vs. young group; *n* = 5. Supplementary Figure 2: establishment of VSMCs replicatively senescence models. (a) Representative Western blot of P16, P21, and P53 in different groups; (b) Quantitative analysis of figure (a); ^∗^*P* < 0.05, ^∗∗^*P* < 0.01, and ^∗∗∗^*P* < 0.001 vs. P3 group. Supplementary Figure 3: establishment of the induced senescence model. The senescence model of VSMCs was established by selecting appropriate concentration and time of glucose stimulation. (a) Representative Western blot of P16, P21, and P53 in different glucose groups; (b) Quantitative analysis of figure (a); (c) Western blot of P16, P21, and P53 protein expressions in VSMCs under 33 mM glucose for indicated time; (d) Quantitative analysis of figure (c); (e) The effect of high glucose on the expressions of GLB1, P16, P21, and P53 detected by Western blot; (f) Quantitative analysis of figure (e). ^∗^*P* < 0.05, ^∗∗^*P* < 0.01, and ^∗∗∗^*P* < 0.001 vs. NG group; #*P* < 0.05 vs. hypertonic (HT) group. Supplementary Figure 4: high glucose stimulated HASMCs to induce cell senescence. (a) Representative *β*-galactosidase staining with the HASMCs (scale bar: 20 *μ*m); (b) Analysis of the relative dyeing rate; (c) Representative Western blot of GLB1, P16, P21, and P53 in different glucose groups; (d) Quantitative different glucose groups; (f) Quantitative analysis of figure (e); ^∗^*P* < 0.05, ^∗∗^*P* < 0.01, and ^∗∗∗^*P* < 0.001 vs. control group. Supplementary Figure 5: effects of high glucose and PGF2*α* stimulation on the FP receptor/Src/PAI-1 pathway and aging-related proteins of HASMCs. (a) Representative Western blot of FP receptor, p-Src, t-Src, and PAI-1 in different groups; (b) Quantitative analysis of figure (a); (c) Representative Western blot of GLB1, P16, and P21 in different groups; (d) Quantitative analysis of figure (e); ^∗^*P* < 0.05 and ^∗∗^*P* < 0.01 vs. NG group. Supplementary Figure 6: effects of inhibition of FP receptor expression in HASMCs on Src/PAI-1 pathways and cell senescence. (a) Representative Western blot of FP receptor, p-Src, t-Src, and PAI-1 in different groups; (b) Quantitative analysis of figure (a); (c) Representative Western blot of GLB1, P16, and P21 in different groups; representative *β*-galactosidase staining with the HASMCs (scale bar: 20 *μ*m); (f) Analysis of the relative dyeing rate; ^∗^*P* < 0.05, ^∗∗^*P* < 0.01, and ^∗∗∗^*P* < 0.001 vs. NG group; #*P* < 0.05 vs. HG Group.

## References

[1] Ungvari Z., Kaley G., de Cabo R., Sonntag W. E., Csiszar A. (2010). Mechanisms of vascular aging: new perspectives. *The Journals of Gerontology. Series A, Biological Sciences and Medical Sciences*.

[2] Ding Y. N., Tang X., Chen H. Z., Liu D. P. (2018). Epigenetic regulation of vascular aging and age-related vascular diseases. *Advances in Experimental Medicine and Biology*.

[3] Qiu H., Zhu Y., Sun Z. (2010). Short communication: vascular smooth muscle cell stiffness as a mechanism for increased aortic stiffness with aging. *Circulation Research*.

[4] Wang J. C., Bennett M. (2012). Aging and Atherosclerosis. *Circulation Research*.

[5] Yu Y., Lucitt M. B., Stubbe J. (2009). Prostaglandin F2alpha elevates blood pressure and promotes atherosclerosis. *Proceedings of the National Academy of Sciences of the United States of America*.

[6] Yavuzer H., Yavuzer S., Cengiz M. (2016). Biomarkers of lipid peroxidation related to hypertension in aging. *Hypertension Research*.

[7] Li Y., Han L., Ding W.-Y. (2015). Prostaglandin F2*α* receptor silencing attenuates vascular remodeling in rats with type 2 diabetes. *Experimental and Molecular Pathology*.

[8] Sales K. J., Boddy S. C., Jabbour H. N. (2008). F-prostanoid receptor alters adhesion, morphology and migration of endometrial adenocarcinoma cells. *Oncogene*.

[9] FAN C., KATSUYAMA M., WEI H., XIA Q., LIU W., YABE-NISHIMURA C. (2010). Molecular mechanisms underlying PGF2alpha-induced hypertrophy of vascular smooth muscle cells. *Yakugaku Zasshi*.

[10] Eren M., Boe A. E., Klyachko E. A., Vaughan D. (2014). Role of plasminogen activator inhibitor-1 in senescence and aging. *Seminars in Thrombosis and Hemostasis*.

[11] Samarakoon R., Higgins S. P., Higgins C. E., Higgins P. J. (2008). TGF-*β*1-induced plasminogen activator inhibitor-1 expression in vascular smooth muscle cells requires pp60^c- _src_^ /EGFR^Y845^ and Rho/ROCK signaling. *Journal of Molecular and Cellular Cardiology*.

[12] Abramovitz M., Boie Y., Nguyen T. (1994). Cloning and expression of a cDNA for the human prostanoid FP receptor.. *The Journal of Biological Chemistry*.

[13] Hutchinson A. J., Coons S. C., Chou C. L. (2010). Induction of angiogenic immediate early genes by activation of FP prostanoid receptors in cultured human ciliary smooth muscle cells. *Current Eye Research*.

[14] Zhang J., Gong Y., Yu Y. (2010). PG F(2*α*) receptor: a promising therapeutic target for cardiovascular disease. *Frontiers in Pharmacology*.

[15] Wong S. L., Leung F. P., Lau C. W. (2009). Cyclooxygenase-2-derived prostaglandin F2alpha mediates endothelium-dependent contractions in the aortae of hamsters with increased impact during aging. *Circulation Research*.

[16] Goupil E., Fillion D., Clément S. (2015). Angiotensin II Type I and Prostaglandin F2*α* Receptors Cooperatively Modulate Signaling in Vascular Smooth Muscle Cells. *The Journal of Biological Chemistry*.

[17] Snetkov V. A., Knock G. A., Baxter L., Thomas G. D., Ward J. P. T., Aaronson P. I. (2006). Mechanisms of the prostaglandin F2alpha-induced rise in [Ca^2+^]i in rat intrapulmonary arteries. *The Journal of Physiology*.

[18] Canugovi C., Stevenson M. D., Vendrov A. E. (2019). Increased mitochondrial NADPH oxidase 4 (NOX4) expression in aging is a causative factor in aortic stiffening. *Redox Biology*.

[19] Gianni D., Bohl B., Courtneidge S. A., Bokoch G. M. (2008). The involvement of the tyrosine kinase c-Src in the regulation of reactive oxygen species generation mediated by NADPH oxidase-1. *Molecular Biology of the Cell*.

[20] Lin C. C., Lee I. T., Yang Y. L., Lee C. W., Kou Y. R., Yang C. M. (2010). Induction of COX-2/PGE_2_/IL-6 is crucial for cigarette smoke extract-induced airway inflammation: Role of TLR4-dependent NADPH oxidase activation. *Free Radical Biology & Medicine*.

[21] Harvey A., Montezano A. C., Lopes R. A., Rios F., Touyz R. M. (2016). Vascular fibrosis in aging and hypertension: molecular mechanisms and clinical implications. *The Canadian Journal of Cardiology*.

[22] Lakatta E. G., Levy D. (2003). Arterial and cardiac aging: major shareholders in cardiovascular disease enterprises: part I: aging arteries: a “set up” for vascular disease. *Circulation*.

[23] Takeshita K., Yamamoto K., Ito M. (2002). Increased expression of plasminogen activator inhibitor-1 with fibrin deposition in a murine model of aging, “klotho” mouse. *Seminars in Thrombosis and Hemostasis*.

[24] Campisi J. (2005). Senescent cells, tumor suppression, and organismal aging: good citizens, bad neighbors. *Cell*.

[25] Vaughan D. E., Rai R., Khan S. S., Eren M., Ghosh A. K. (2017). Plasminogen activator inhibitor-1 is a marker and a mediator of senescence. *Arteriosclerosis, Thrombosis, and Vascular Biology*.

[26] Elzi D. J., Lai Y., Song M., Hakala K., Weintraub S. T., Shiio Y. (2012). Plasminogen activator inhibitor 1--insulin-like growth factor binding protein 3 cascade regulates stress-induced senescence. *Proceedings of the National Academy of Sciences of the United States of America*.

