# Supplementary material for: PGF2α-FP Receptor Ameliorates Senescence of VSMCs in Vascular Remodeling by Src/PAI-1 Signal Pathway
Source: Oxid Med Cell Longev. 2022 Jan 27;2022:2908261. doi: 10.1155/2022/2908261 (PMC8813271; doi:10.1155/2022/2908261)
Supplement: Supplementary Materials — Supplementary Figure 1: establishment of natural aging mouse model. WT C57 mice were divided into young and old groups. (a) Representative Western blot of P16, P21, and P53; (b) Western blot analysis of P16, P21, and P53; (c) Representative β-galactosidase staining with the aorta from the mice (scale bar: 20 μm); (d) Analysis of the relative dyeing rate. ∗P < 0.05, ∗∗P < 0.01, and ∗∗∗P < 0.001 vs. young group; n = 5. Supplementary Figure 2: establishment of VSMCs replicatively senescence models. (a) Representative Western blot of P16, P21, and P53 in different groups; (b) Quantitative analysis of figure (a); ∗P < 0.05, ∗∗P < 0.01, and ∗∗∗P < 0.001 vs. P3 group. Supplementary Figure 3: establishment of the induced senescence model. The senescence model of VSMCs was established by selecting appropriate concentration and time of glucose stimulation. (a) Representative Western blot of P16, P21, and P53 in different glucose groups; (b) Quantitative analysis of figure (a); (c) Western blot of P16, P21, and P53 protein expressions in VSMCs under 33 mM glucose for indicated time; (d) Quantitative analysis of figure (c); (e) The effect of high glucose on the expressions of GLB1, P16, P21, and P53 detected by Western blot; (f) Quantitative analysis of figure (e). ∗P < 0.05, ∗∗P < 0.01, and ∗∗∗P < 0.001 vs. NG group; #P < 0.05 vs. hypertonic (HT) group. Supplementary Figure 4: high glucose stimulated HASMCs to induce cell senescence. (a) Representative β-galactosidase staining with the HASMCs (scale bar: 20 μm); (b) Analysis of the relative dyeing rate; (c) Representative Western blot of GLB1, P16, P21, and P53 in different glucose groups; (d) Quantitative different glucose groups; (f) Quantitative analysis of figure (e); ∗P < 0.05, ∗∗P < 0.01, and ∗∗∗P < 0.001 vs. control group. Supplementary Figure 5: effects of high glucose and PGF2α stimulation on the FP receptor/Src/PAI-1 pathway and aging-related proteins of HASMCs. (a) Representative Western blot of FP receptor, p-Src, [file 2908261.f1.pdf]

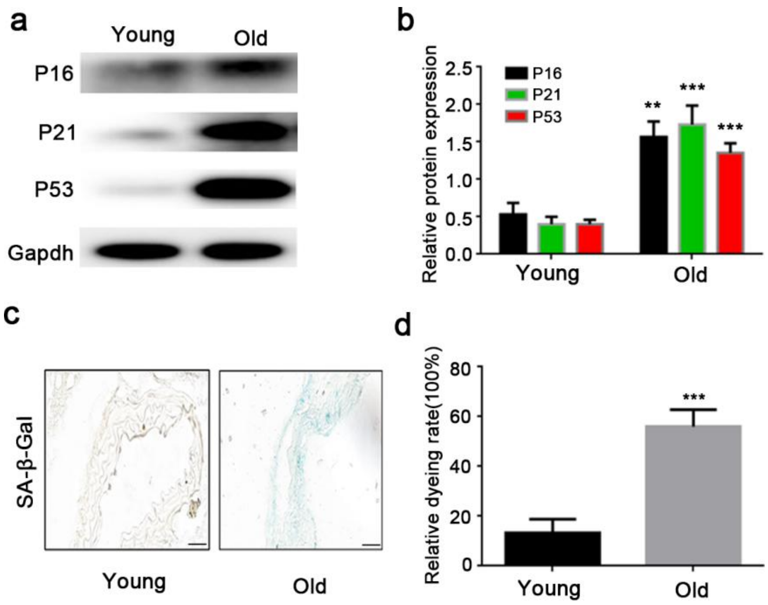

Supplementary Figure.1 Establishment of natural aging mice model. WT C57 mice were divided into young and old group. (a) Representative Western blot of P16, P21, and P53; (b) Western blot analysis of P16, P21, and P53; (c) Representative β-galactosidase staining with the aorta from the mice (scale bar: 20μm); (d) Analysis of the relative dyeing rate. **P*<0.05, ***P*<0.01 and ****P*<0.001 vs Young Group; n=5.

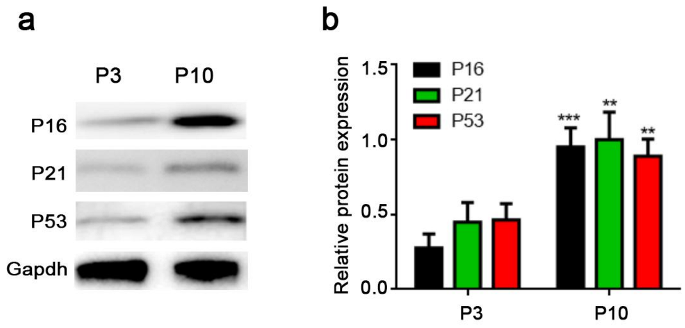

Supplementary Figure.2 Establishment of VSMCs replicatively senescence models. (a) Representative Western blot of P16, P21, and P53 in different groups; (b) Quantitative analysis of Figure a; *$P<0.05$, **$P<0.01$ and ***$P<0.001$ vs P3 Group.

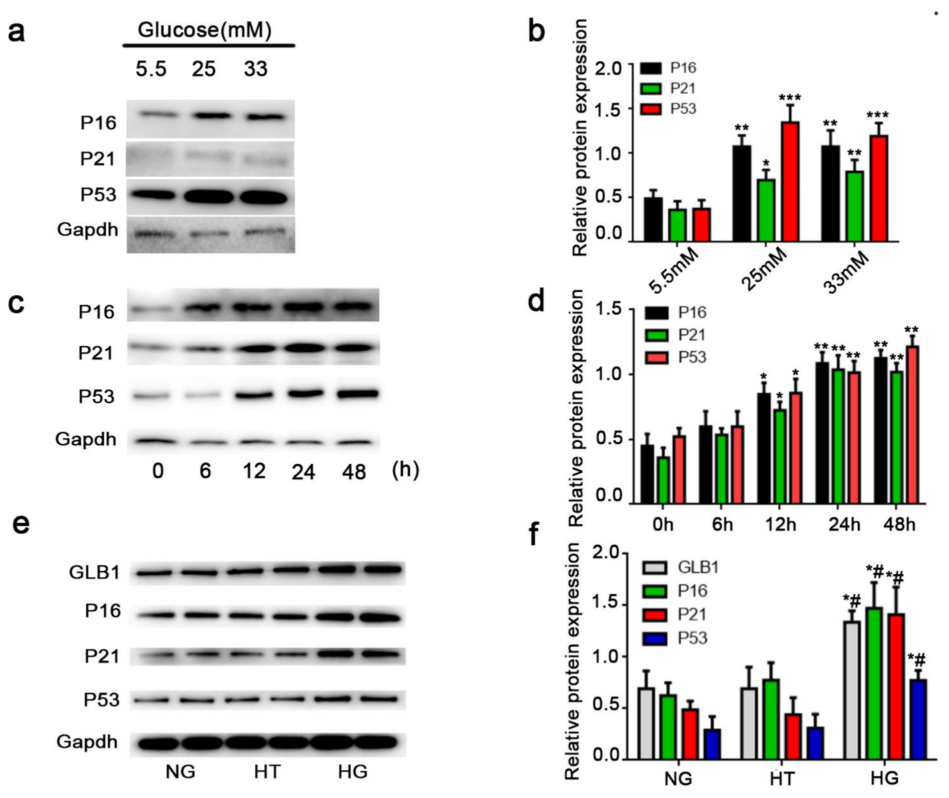

Supplementary Figure.3 Establishment of induced senescence model. The senescence model of VSMCs was established by selecting appropriate concentration and time of glucose stimulation. (a) Representative Western blot of P16, P21, and P53 in different glucose groups; (b) Quantitative analysis of Figure a; (c) Western blot of P16, P21, and P53 protein expressions in VSMCs under 33mM glucose for indicated time; (d) Quantitative analysis of Figure c; (e) The effect of high glucose on the expressions of GLB1, P16, P21 and P53 detected by Western bolt; (f) Quantitative analysis of Figure e. **P*<0.05, ***P*<0.01, ****P*<0.001 vs NG Group; $^{\#}P<0.05$ vs Hypertonic (HT) Group.

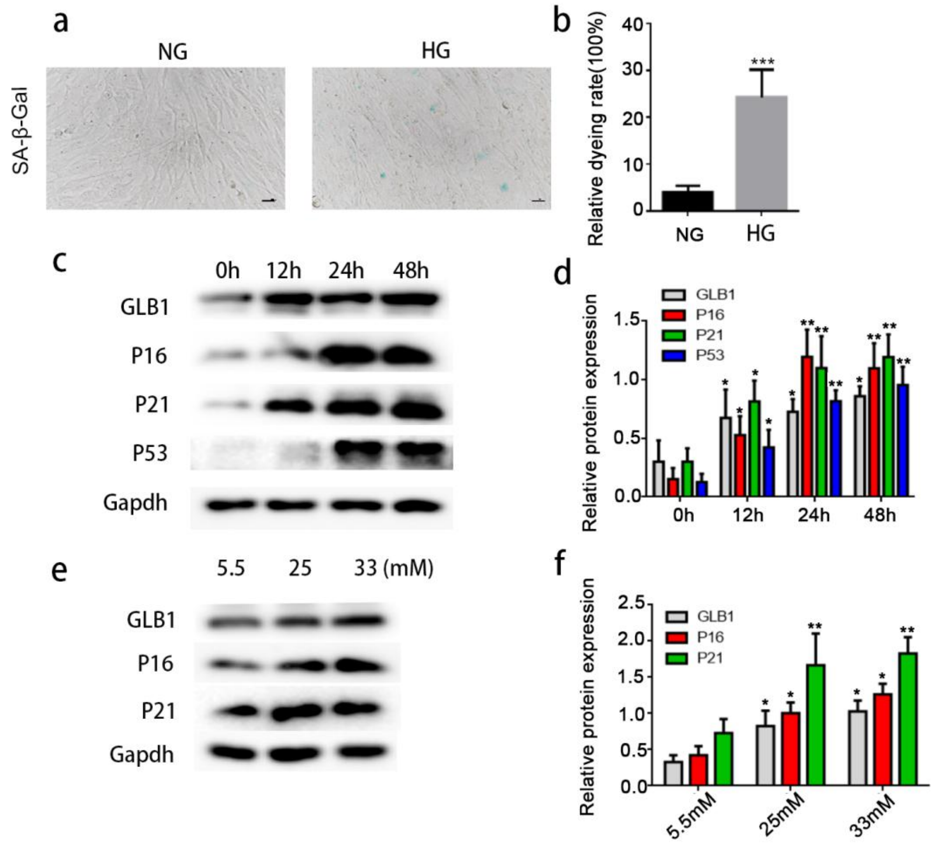

Supplementary Figure.4 High glucose stimulated HASMCs to induce cell senescence. (a) Representative β-galactosidase staining with the HASMCs (scale bar: 20μm); (b) Analysis of the relative dyeing rate; (c) Representative Western blot of GLB1, P16, P21, and P53 in different glucose groups; (d) Quantitative

different glucose groups; (f) Quantitative analysis of Figure e; **P*<0.05, ***P*<0.01 and ****P*<0.001 vs Control Group.

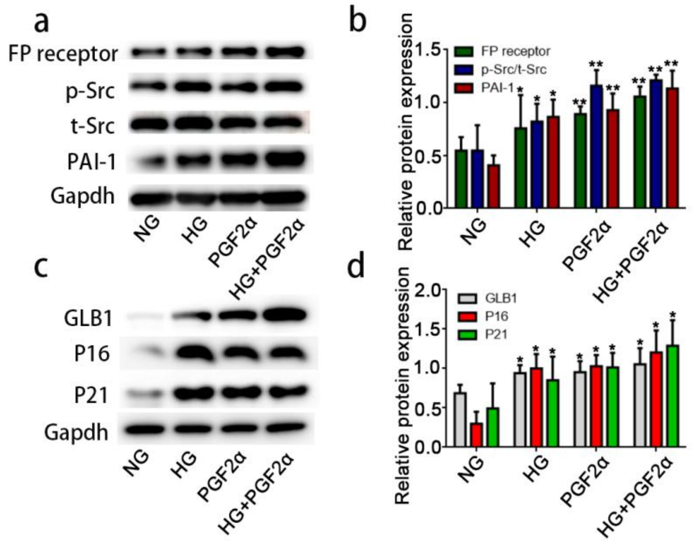

Supplementary Figure.5 Effects of high glucose and PGF2α stimulation on the FP recpetor/Src/PAI-1 pathway and aging-related proteins of HASMCs. (a) Representative Western blot of FP receptor, p-Src, t-Src, PAI-1 in different groups; (b) Quantitative analysis of Figure a; (c) Representative Western blot of GLB1, P16 and P21 in different groups; (d) Quantitative analysis of Figure e; **P*<0.05 and ***P*<0.01 vs NG Group.

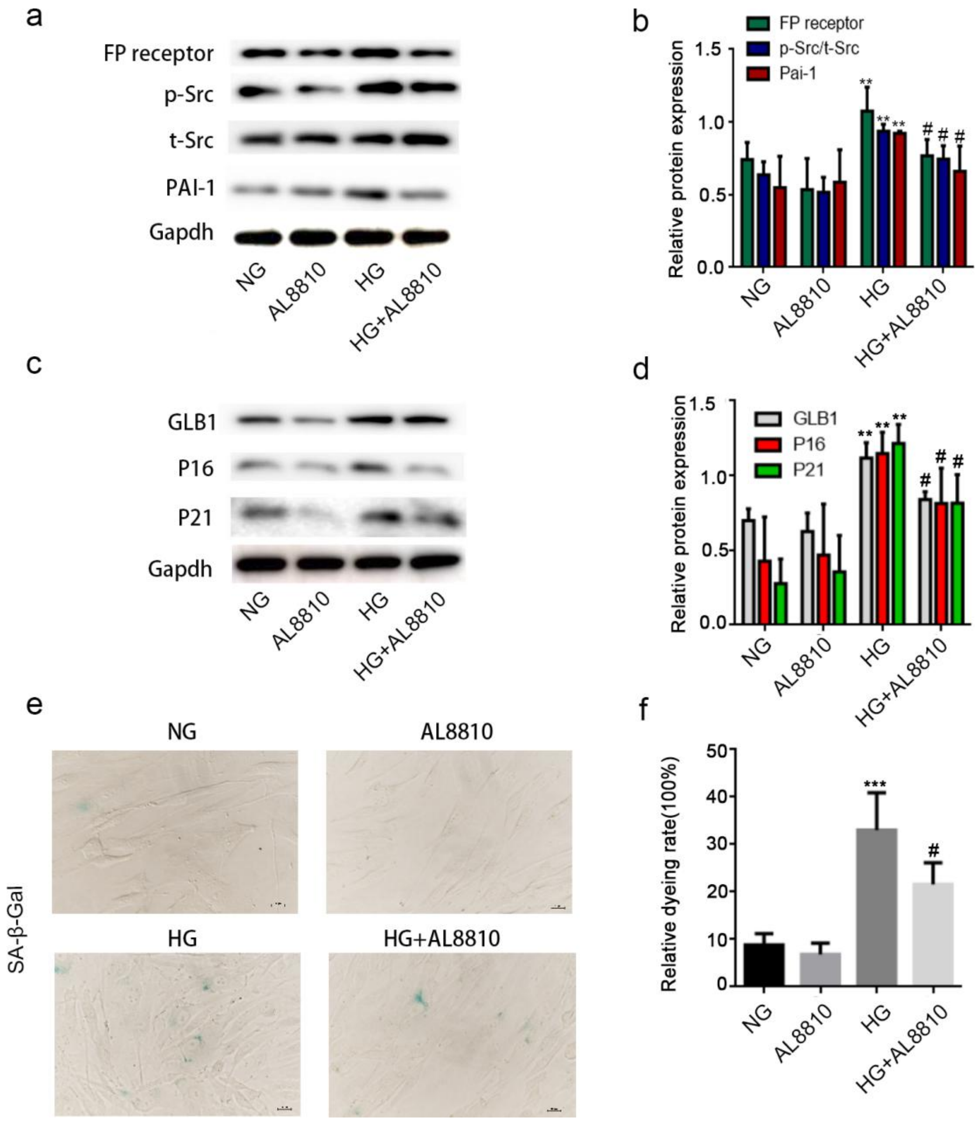

Supplementary Figure.6 Effects of inhibition of FP receptor expression in HASMCs on Src/PAI-1 pathways and cells senescence. (a) Representative Western blot of FP receptor, p-Src, t-Src, PAI-1 in different groups; (b) Quantitative analysis of Figure a; (c) Representative Western blot of GLB1, P16 and P21 in different groups; Representative β-galactosidase staining with the HASMCs (scale bar: 20μm); (f) Analysis of the relative dyeing rate; **P*<0.05, ***P*<0.01 and ****P*<0.001 vs NG Group ; $^{\#}$*P*<0.05 vs HG Group.
